# Supplementary material for: R2TP/Prefoldin-like component RUVBL1/RUVBL2 directly interacts with ZNHIT2 to regulate assembly of U5 small nuclear ribonucleoprotein
Source: Nat Commun. 2017 May 31;8:15615. doi: 10.1038/ncomms15615 (PMC5460035; doi:10.1038/ncomms15615)
Supplement: Supplementary Information — Supplementary Figures and Supplementary Table [file ncomms15615-s1.pdf]

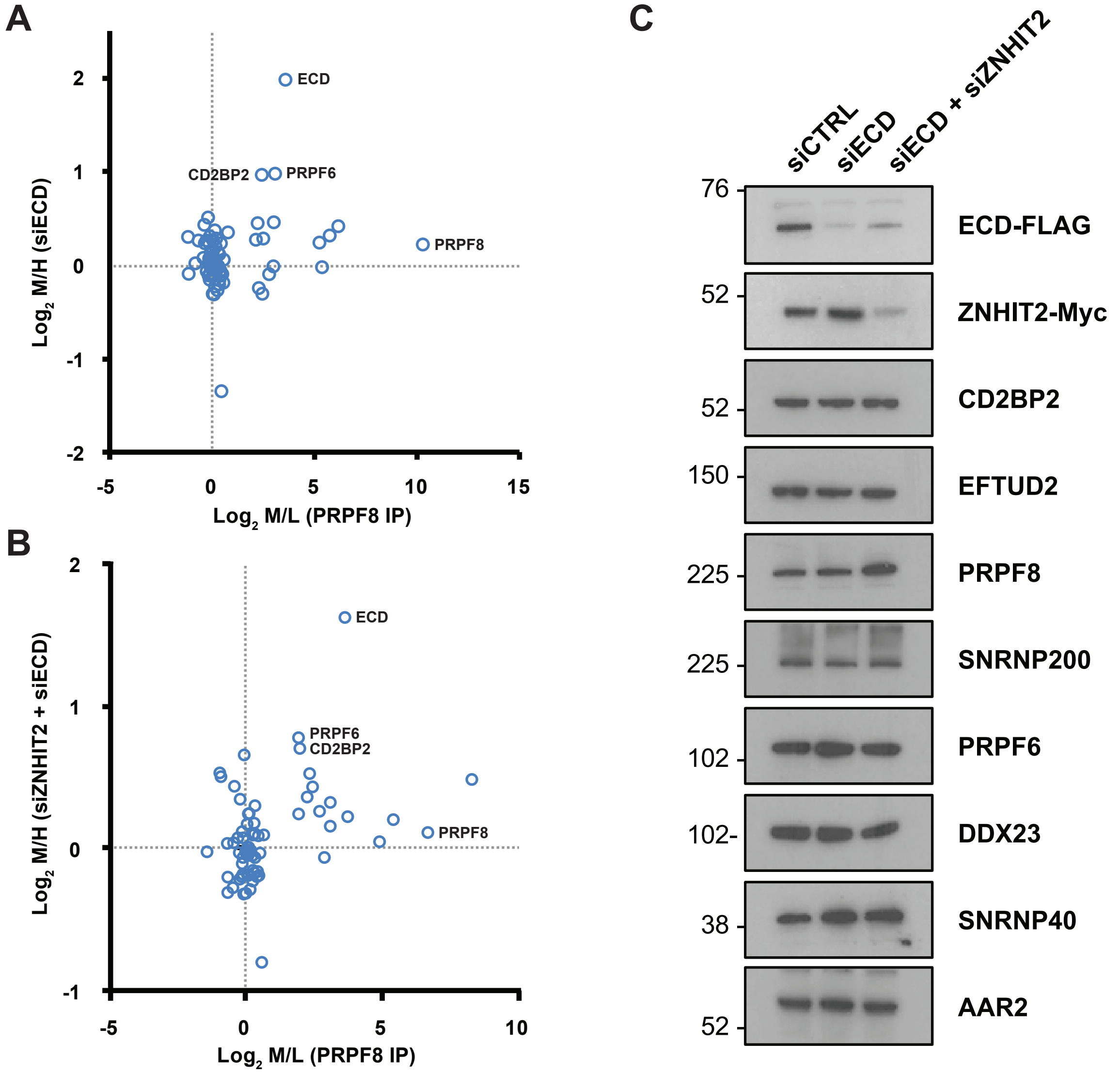

Supplementary Figure 1. ECD also regulates the composition of the U5 snRNP

(A-B) Interpretation of the SILAC data on scatterplots. Intensity ratio of medium-labeled proteins over unlabeled proteins (M/L) on the X axis denotes IP enrichment. Intensity ratio of medium-labeled proteins over heavy-labeled proteins (M/H) on the Y axis represents experimental variations. Marked proteins in the upper right quadrant are proteins enriched in the IP that were less abundant following siRNA treatment. Position of the protein used for IP is shown in all experiments. (C) Expression assessment of various U5 snRNP subunits following treatment with either siECD or both siECD and siZNHIT2

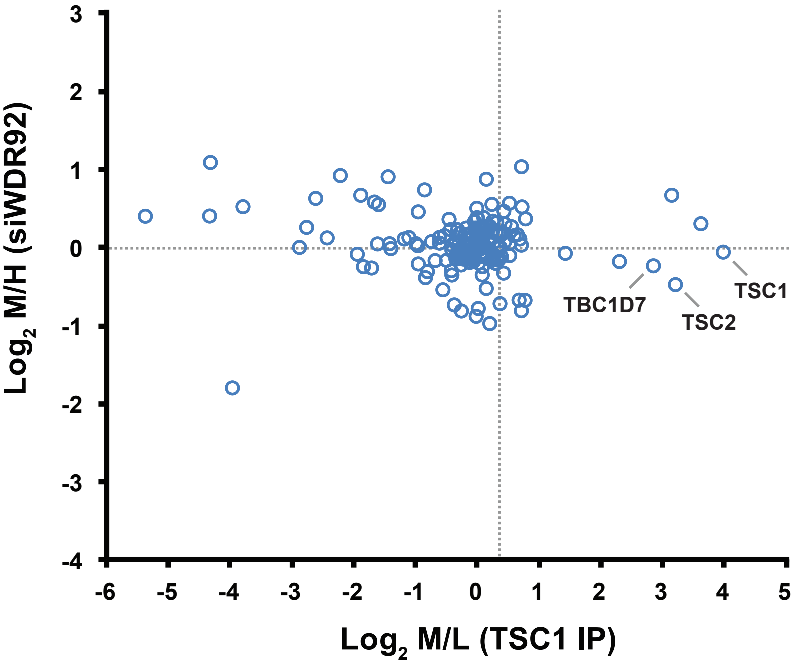

Supplementary Figure 2. Knockdown of R2TP subunit WDR92 does not appear to regulate the composition of the TSC1-TSC1 complex

Intensity ratio of medium-labeled proteins over unlabeled proteins (M/L) on the X axis denotes IP enrichment (mediated by TSC1-FLAG). Intensity ratio of medium-labeled proteins over heavy-labeled proteins (M/H) on the Y axis represents experimental variations. Marked proteins show the three subunits of the TSC1-TSC2 complex that were enriched in the IP but whose abundance was not significantly affected by siRNA treatment.

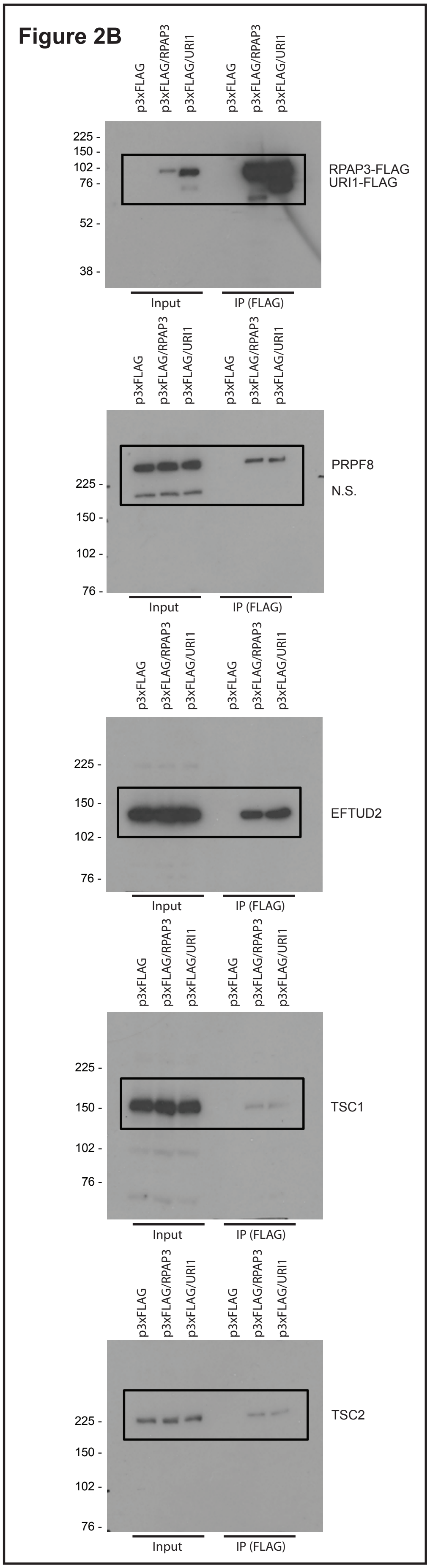

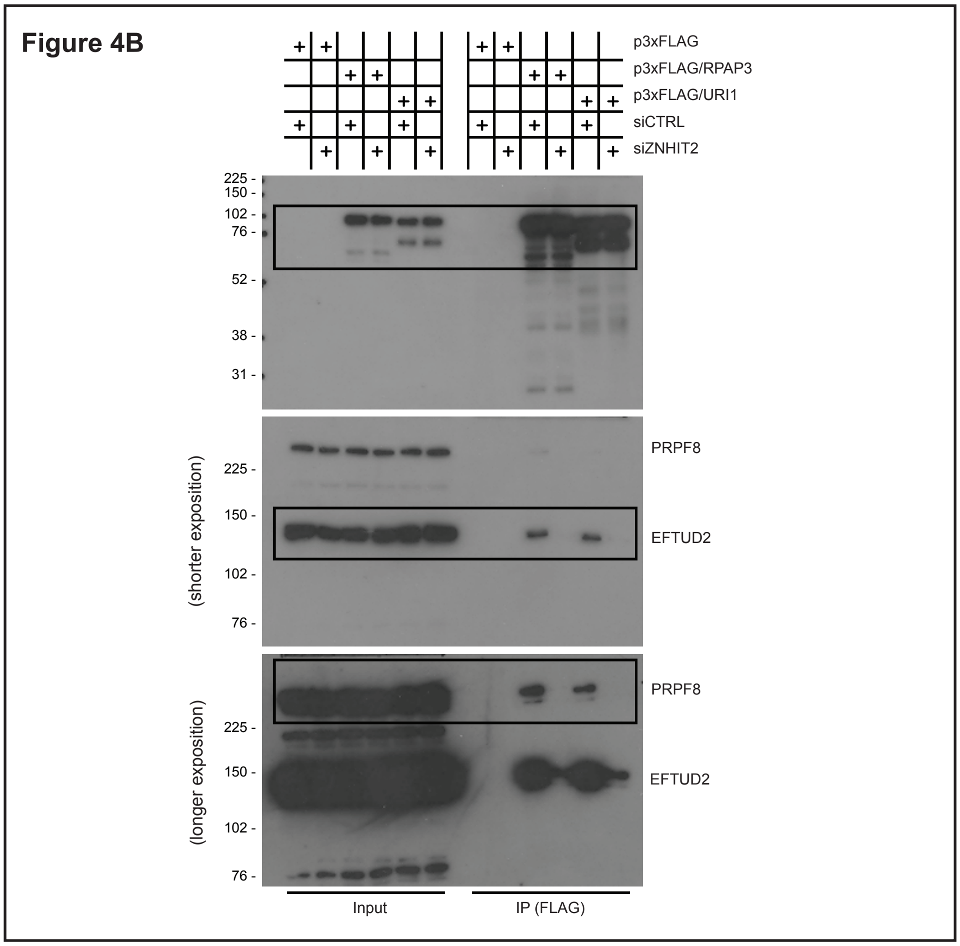

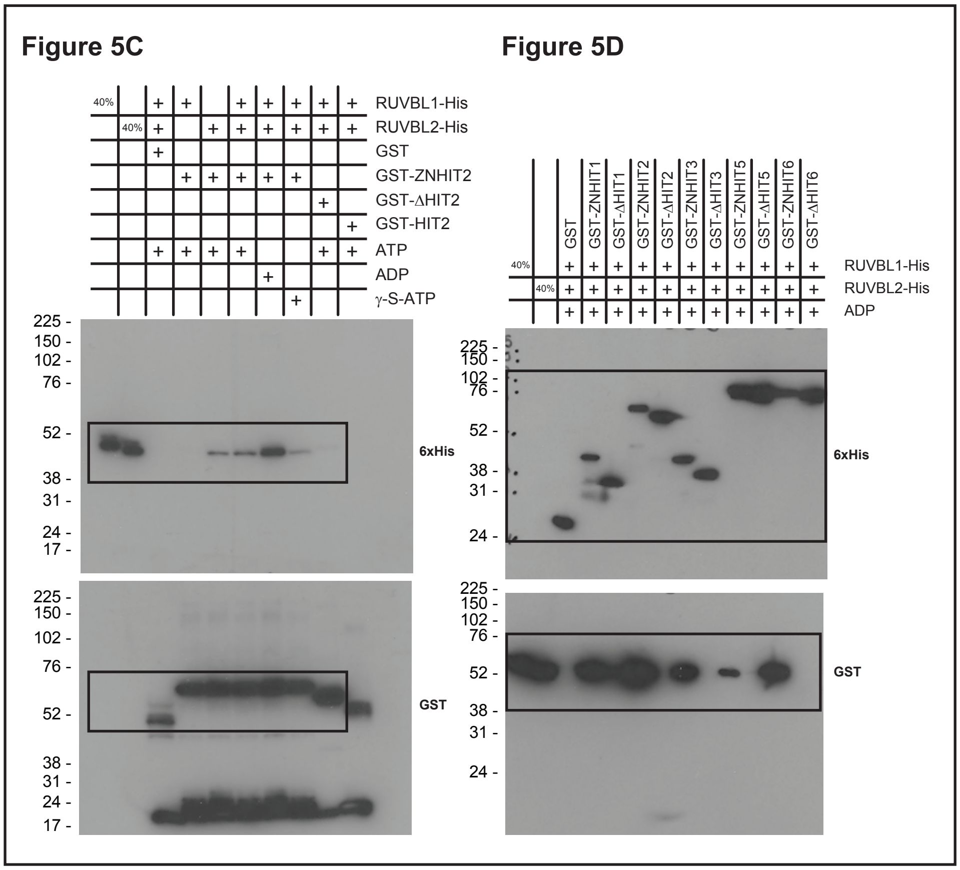

Supplementary Figure 3. Uncropped scans of immuno-blots shown in the figures

Boxes indicate the cropped areas used in the figure.

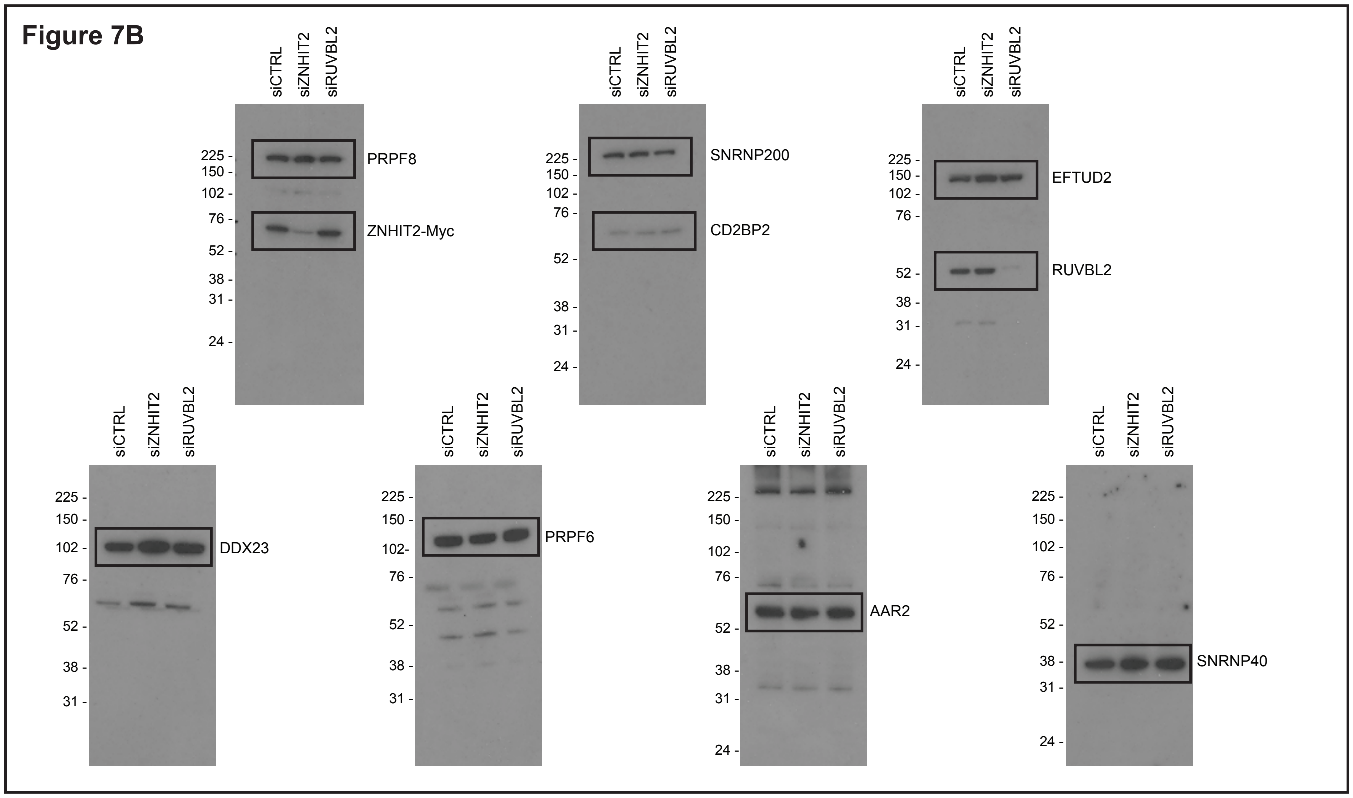

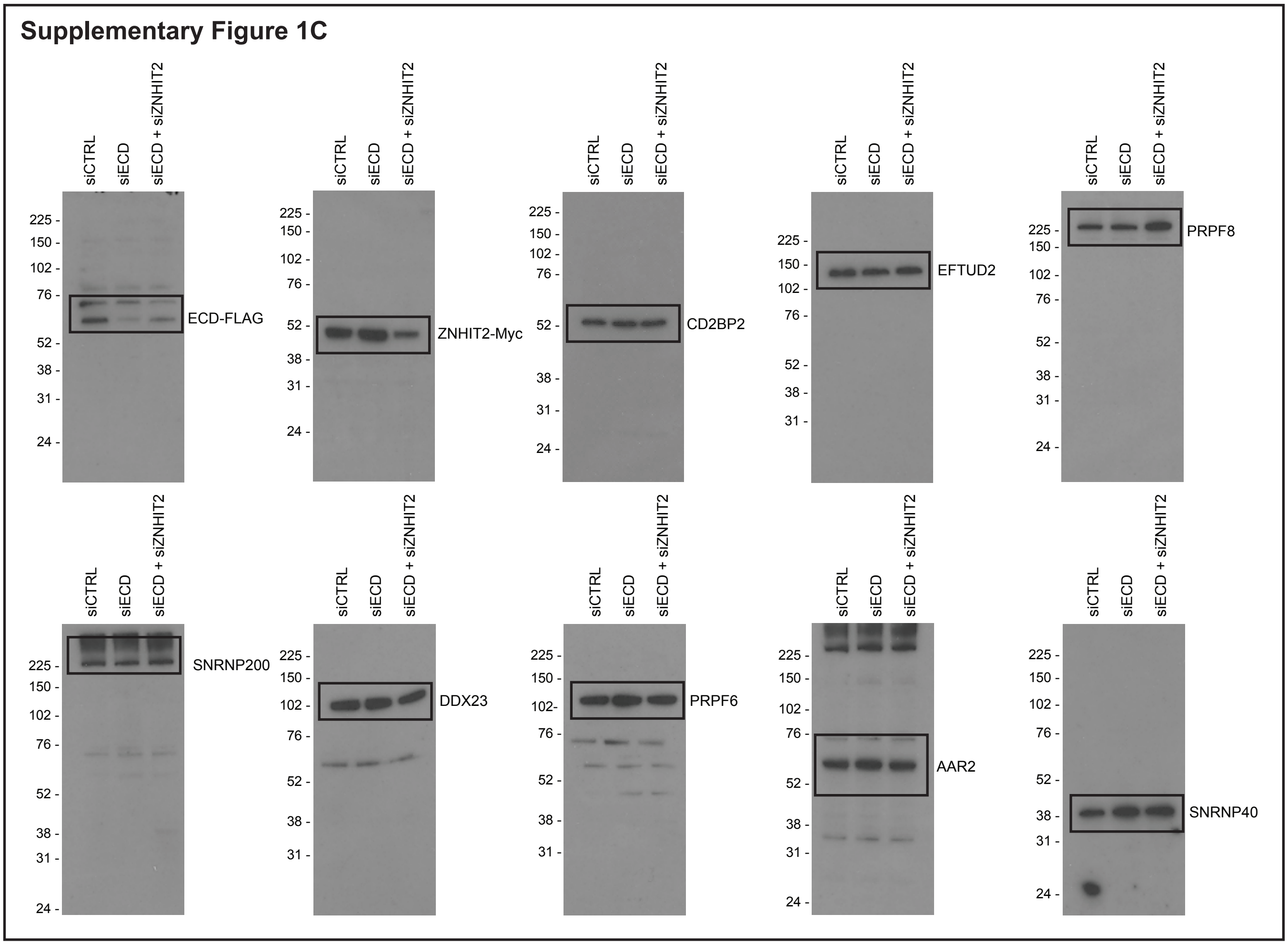

Supplementary Figure 4. Uncropped scans of immuno-blots shown in the figures
Boxes indicate the cropped areas used in the figure.

Vector	cDNA	Source	5' end	3' end
pMZI	AAR2	IMAGE 3536406	XhoI	NotI
pMZI	CD2BP2	IMAGE 2820690	XhoI	NdeI
pMZI	EAPP	IMAGE 3460193	XhoI	NdeI
pMZI	ECD	IMAGE 3504956	XbaI	NotI
pMZI	FBXO3	IMAGE 5549548	XbaI	NotI
pMZI	NCDN	IMAGE 3914058	XhoI	NdeI
pMZI	POLDIP2	IMAGE 3943941	XhoI	NdeI
pMZI	RPAP3	IMAGE 5218249	NotI	NdeI
pMZI	RUVBL1	IMAGE 591284	NotI	NdeI
pMZI	RUVBL2	IMAGE 3051517	NotI	NdeI
pMZI	SLC7A6OS	IMAGE 4345035	XbaI	NdeI
pMZI	TBC1D7	IMAGE 3828592	XhoI	NdeI
modified pMZI	TSC1	pRK7-FLAG-TSC1	EcoRV	NotI
pMZI	TSC2	pRK7-FLAG-TSC2	XhoI	NdeI
pMZI	TSSC4	IMAGE 3677524	XhoI	NdeI
pMZI	URI1	IMAGE 6500727	XhoI	NdeI
pMZI	WDR92	IMAGE 6164689	XhoI	NotI
pMZI	ZNHIT1	LIFESEQ 1268277	XhoI	NdeI
pMZI	ZNHIT2	IMAGE 6107352	XhoI	NdeI
pMZI	ZNHIT3	LIFESEQ 7179220	XhoI	NdeI
pMZI	INO80B	IMAGE 5760624	XhoI	NdeI
pMZI	DDX59	IMAGE 5302098	XhoI	NdeI
pMZI	ZNHIT6	IMAGE 6012059	XhoI	NdeI
pDEST-pcDNA5-BirA-FLAG C-term	ZNHIT2	IMAGE 6107352	Gateway	
p3xFLAG-CMV-14	ECD	IMAGE 3504956	NotI	XbaI
p3xFLAG-CMV-14	EFTUD2	IMAGE 2960986	NotI	XbaI
p3xFLAG-CMV-14	PRPF8	IMAGE 5587081	NotI	XbaI
p3xFLAG-CMV-14	RPAP3	IMAGE 5218249	NotI	XbaI
p3xFLAG-CMV-14	URI1	IMAGE 6500727	NotI	XbaI
pcDNA3-Myc	ZNHIT2	IMAGE 6107352	EcoRI	XhoI
pcDNA3-Myc	ΔHIT2	IMAGE 6107352	EcoRI	XhoI
pET-23a+	RUVBL1	IMAGE 591284	BamHI	XhoI
pET-23a+	RUVBL2	IMAGE 3051517	BamHI	XhoI
pGEX-4T-1	ZNHIT1	LIFESEQ 1268277	SalI	NotI
pGEX-4T-1	ΔHIT1	LIFESEQ 1268277	SalI	NotI
pGEX-4T-1	ZNHIT2	IMAGE 6107352	BamHI	XhoI
pGEX-4T-1	ΔHIT2	IMAGE 6107352	BamHI	XhoI
pGEX-4T-1	ZNHIT3	LIFESEQ 7179220	SalI	NotI
pGEX-4T-1	ΔHIT3	LIFESEQ 7179220	SalI	NotI
pGEX-4T-1	INO80B	IMAGE 5760624	SalI	NotI
pGEX-4T-1	ΔHIT4	IMAGE 5760624	SalI	NotI
pGEX-4T-1	DDX59	IMAGE 5302098	SalI	NotI
pGEX-4T-1	ΔHIT5	IMAGE 5302098	SalI	NotI
pGEX-4T-1	ZNHIT6	IMAGE 6012059	SalI	NotI
pGEX-4T-1	ΔHIT6	IMAGE 6012059	SalI	NotI

Supplementary Table 1. List of expression vectors created for this manuscript and cloning strategy
